# Review of “*Mathematical Models for Neglected Tropical Diseases: Essential Tools for Control and Elimination, Part B*” Edited by Maria-Gloria Basáñez and Roy M. Anderson

**DOI:** 10.1186/s13071-017-1974-2

**Published:** 2017-01-21

**Authors:** Robert C. Spear

**Affiliations:** School of Public Health, 50 University Hall, University of California, Berkeley, 94720-7360 CA USA

## Abstract

Basáñez MG, Anderson RM, Editors: *Mathematical Models for Neglected Tropical Diseases: Essential Tools for Control and Elimination, Part B,* *Volume 94*, *Advances in Parasitology*, Academic Press; 2016, 430 pages. ISBN: 978-0-12-809971-1

## Review

This volume is a complement to earlier collections addressing mathematical modelling of neglected tropical diseases principally from participants in the London Centre for Neglected Tropical Disease Research (LCNTDR) and the NTD Modelling Consortium. However, readers unfamiliar with that literature will find the volume a nicely balanced collection of chapters on modelling the transmission of NTDs, ranging from bacterial infections to helminths, but principally directed at those with substantial background in infectious diseases, modelling of biological systems, or at their intersection.

The volume begins with a well written chapter by Pinsent et al. on trachoma transmission. After a brief introduction to the disease, the chapter is based on a comprehensive review of the literature, followed by an assessment of the strengths and weaknesses of models in understanding transmission and informing control programmes. Of the five diseases addressed in this volume, one is left with the impression that the modelling of trachoma transmission has had the least impact on control programmes, an issue addressed in the chapter.

While there are several themes common to most chapters, the five disease-specific chapters provide instructive contrasts in the challenge of developing useful models given the differences in current understanding of the biology of this group of diseases. Chapter 4 on schistosomiasis modelling and control by Anderson and colleagues lies at one end of this spectrum with a succinct review of the transmission processes followed by a review and updating of the classic Anderson-May model. The focus here is on using the models to study mass drug administration (MDA). At the other end of the spectrum is Chapter 2 on visceral leishmaniasis by Rock and colleagues. The opening sentence of their section on diseases in humans lays out one dimension of the challenge: “Leishmaniasis is characterized by a diversity of aetiological agents causing cutaneous (CL), mucocutaneous (MCL) and visceral (VL) presentations dependent on the species or strain of *Leishmania* …” Moreover, the infectivity of individuals with clinical disease to the sandfly vector, or that of those infected but asymptomatic, is unclear. Hence, this chapter has a 20 page introduction to the disease and its complexities prior to addressing the current state of modelling transmission.

The chapter on soil-transmitted helminths by Truscott et al. is also an application of the Anderson-May framework aimed at assessing the impact of MDA on disease control and criteria for transmission elimination in particular. Similarly, the modelling section of Chapter 5 by Basáñez et al. on river blindness begins with a review of the EPIONCHO model that is also in the Anderson-May tradition. In contrast with Chapters 3 and 4 which are focused on MDA, vector control was more prominently addressed in the modelling of onchocerciasis from early in model development, no doubt due to interest in vector control in control activities. That history is summarized and is of general interest, but the core of the chapter is mainly addressed to aficionados and comprises a detailed discussion and comparison of the population-based EPIONCHO and the individual-based model ONCHOSIM. ONCHOSIM, however, is also of broader interest as a pioneering application of individual or agent-based models to infectious disease transmission, an approach now being utilized by many others.

Two themes that run through all of the disease-specific chapters relate to the importance and challenges of infection diagnosis on one hand and the estimation of model parameters on the other. Chapter 6 by Medley et al. addresses diagnostics in the case of helminths utilizing an individual-based model to explore the impact of diagnostic sensitivity on MDA campaigns for the control and elimination of *Ascaris lumbricoides.* Notably, disability-adjusted life years, DALYs, are used as a performance metric. The chapter serves as an instructive example of the interaction between diagnostic sensitivity, baseline prevalence and the nature and characteristics of the intervention.

Parameter estimation is a major challenge in many if not most applications of complex mathematical models due to the inherently large number of parameters in these models coupled with the paucity of data generally available to inform their values. There is a substantial literature on this subject in other areas of application that is not addressed in this volume, but the challenge remains formidable. It is most succinctly laid out in this volume in the section of Chapter 4 on parameter estimation.

The final chapter by Kastner et al. provides a fitting closure in addressing renewed interest in the planning of disease eradication campaigns in the context of an “eradication investment case” including a useful discussion of criteria for elimination. The focus is on lymphatic filariasis and the authors’ use of the EPIFIL model to explore the feasibility of eradication relying principally on MDA. This chapter also illustrates that, although much progress has been made in bringing quantitative methods to the planning and analysis of infectious disease control programmes, much remains to be done.

## Abbreviations

DALY: Disability-adjusted life years
MDA: Mass drug administration
NTD: Neglected tropical diseases

